# Hyaluronic Acid Gel-Based Scaffolds as Potential Carrier for Growth Factors: An In Vitro Bioassay on Its Osteogenic Potential

**DOI:** 10.3390/jcm5120112

**Published:** 2016-11-30

**Authors:** Masako Fujioka-Kobayashi, Benoit Schaller, Eizaburo Kobayashi, Maria Hernandez, Yufeng Zhang, Richard J. Miron

**Affiliations:** 1 Department of Cranio-Maxillofacial Surgery, Inselspital, Bern University Hospital, Bern 3010, Switzerland; benoit.schaller@insel.ch (B.S.); eizabu@ngt.ndu.ac.jp (E.K.); 2 Department of Oral Surgery, Institute of Biomedical Sciences, Tokushima University Graduate School, Tokushima 770-8501, Japan; masako@tokushima-u.ac.jp; 3 Department of Periodontology, Nova Southeastern University, Fort Lauderdale, FL 33328, USA; marher@nova.edu; 4 Department of Oral Implantology, University of Wuhan, Wuhan 430079, China; zyf@whu.edu.cn; 5 Cell Therapy Institute, Center for Collaborative Research, Nova Southeastern University, Fort Lauderdale, FL 33328, USA; rmiron@nova.edu; 6 Department of Periodontics and Oral Medicine, University of Michigan School of Dentistry, Ann Arbor, Michigan, MI 48109-1078, USA; rmiron@umich.edu

**Keywords:** osteoinduction, osteoinductive, guided bone regeneration, bone formation, bone induction, BMP, growth factor, dimensional changes, regenerative therapy, hard tissue regeneration

## Abstract

Hyaluronic acid (HA) has been utilized for a variety of regenerative medical procedures due to its widespread presence in connective tissue and perceived biocompatibility. The aim of the present study was to investigate HA in combination with recombinant human bone morphogenetic protein 9 (rhBMP9), one of the most osteogenic growth factors of the BMP family. HA was first combined with rhBMP9 and assessed for the adsorption and release of rhBMP9 over 10 days by ELISA. Thereafter, ST2 pre-osteoblasts were investigated by comparing (1) control tissue culture plastic, (2) HA alone, and (3) HA with rhBMP9 (100 ng/mL). Cellular proliferation was investigated by a MTS assay at one, three and five days and osteoblast differentiation was investigated by alkaline phosphatase (ALP) activity at seven days, alizarin red staining at 14 days and real-time PCR for osteoblast differentiation markers. The results demonstrated that rhBMP9 adsorbed within HA scaffolds and was released over a 10-day period in a controlled manner. While HA and rhBMP9 had little effect on cell proliferation, a marked and pronounced effect was observed for cell differentiation. rhBMP9 significantly induced ALP activity, mRNA levels of collagen1α2, and ALP and osteocalcin (OCN) at three or 14 days. HA also demonstrated some ability to induce osteoblast differentiation by increasing mRNA levels of OCN and increasing alizarin red staining at 14 days. In conclusion, the results from the present study demonstrate that (1) HA may serve as a potential carrier for various growth factors, and (2) rhBMP9 is a potent and promising inducer of osteoblast differentiation. Future animal studies are now necessary to investigate this combination approach in vivo.

## 1. Introduction

Bone morphogenetic proteins (BMPs) have played a pivotal role in modern medicine by directly influencing the commitment and differentiation of osteoprogenitor cells towards osteoblasts [1]. When combined with various tissue engineering strategies, they guide the induction of mesenchymal progenitor cells to differentiate towards bone-forming osteoblasts. As it relates to clinical practice, recombinant human (rh)BMP2 has been the most widely utilized BMP having been used for a variety of clinical procedures including spinal fusion, open tibial fractures and various bone augmentation procedures in regenerative dentistry [2,3,4]. Despite this, it remains surprising that of all the 15 BMPs, BMP2 is not necessarily the most osteoinductive of the BMP family [5].

Over a decade ago, two pioneering studies investigated and directly compared the regenerative potential of 14 BMPs via adenovirus transfection experiments (gene therapy) [5,6]. Cheng et al. demonstrated that alkaline phosphatase activity (a marker for osteoblast differentiation) was highest in BMP9 whereas Kang et al. reported that both BMP-6 and -9 had greater in vivo potential for ectopic bone formation [5,6]. Since those studies utilized adenovirus transfection experiments (an area of research still not approved by the food and drug administration (FDA) [7], translating these results into a clinical setting has not been attempted.

BMP9 (also known as growth differentiation factor 2; GDF2) was first identified in 1995 in the developing mouse liver cDNA libraries [8]. Since then, BMP9 has been shown to play a role in many pathways including osteogenesis, angiogenesis and chondrogenesis [9,10,11]. One of the drawbacks of these few studies characterizing BMP9 were that they were only performed utilizing adenovirus transfections (gene therapy) with no information regarding its recombinant protein activity [5,6,10,11]. Recently our research group investigated for the first time the regenerative potential of a clinically viable recombinant source of human (rh)BMP9 [12,13]. In two separate studies, it was found that rhBMP9 demonstrated up to 10 times more osteopromotion in in vitro osteoblast differentiation when compared to rhBMP2 [12,13].

Critical to the success of tissue engineering strategies utilizing growth factors are their biomaterial carrier systems [14]. While the adsorption of BMPs to bone biomaterials has been a highly studied topic in recent years [15,16,17,18], additional strategies designed to facilitate the delivery of growth factors remain necessary. Hyaluronic acid (HA) has been utilized in recent years in various medical fields due to its natural constitution in human connective tissues. It is an anionic, non-sulfated glycosaminoglycan considered an optimal biomaterial for tissue engineering, with inherent biocompatible and bioresorbable properties [16]. It also plays a prominent role as a treatment agent for various medical conditions including chronic osteoarthritis, aesthetic surgery, dermatology, ophthalmology, oral maxilla-facial surgery, as well as for various tissue engineering applications [17,18,19,20]. HA is also available in cross-linked forms for various tissue engineering applications serving as a scaffold to further improve the overall mechanical properties of the scaffolding system [21,22,23,24].

Therefore, in light of the previous utilization of HA in its cross-linked format as a tissue engineering scaffold, the aim of the present study was to determine whether HA could also serve as a carrier for regenerative growth factors such as rhBMP9, one of the most osteogenic growth factors known to date. First, the growth factor release of rhBMP9 over time was investigated from a zero- to 10-day period within HA scaffolds. Thereafter, pre-osteoblasts were quantified for their ability to attach, proliferate and differentiate on HA scaffolds with/without rhBMP9.

## 2. Methods

### 2.1. Hyaluronic Acid and Recombinant Human BMP9

**Figure 1:**
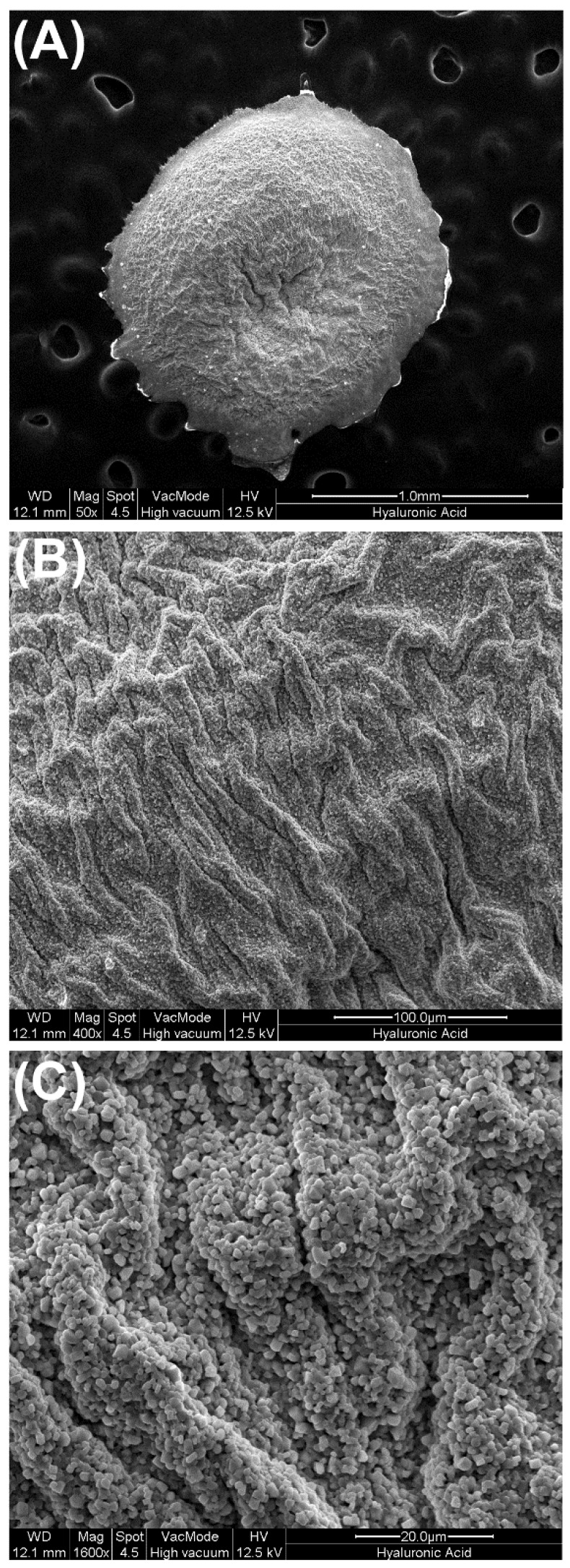
Scanning electron microscopy (SEM) of hyaluronic acid scaffolds at (**A**) low and (**B**) high magnification. Notice the roughened surface topography of the cross-linked HA apparent at very high magnification (**C**).

Recombinant human (rh)BMP9 was purchased from R&D systems Inc (Minneapolis, MM, USA). Hyaluronic acid (HA) was kindly provided by Regedent (Zürich, Switzerland) utilizing a carrier system including cross-linked HA (16 mg HA/mL—cross-linked at 1MDa; Hyadent BG, BioScience GmbH, Ransbach-Baumbach, Germany, crosslinked to butanediol diglycidyl ether (BDDE)). Figure 1 demonstrates a scanning electron microscopy (SEM) image used to characterize surface shape and topography as previously described [25]. For all in vitro experiments, the following three groups were utilized: (1) control standard tissue culture plastic (TCP), (2) control HA alone and (3) HA with 100 ng/mL of rhBMP9. Undifferentiated mouse cell-line ST2 was obtained from RIKEN Cell Bank (Tsukuba, Japan) and therefore no ethical approval was necessary for the present study. Cells were cultured in a humidified atmosphere at 37 °C in growth medium consisting of DMEM (Invitrogen Corporation, Carlsbad, CA, USA), 10% fetal Bovine serum (FBS; Invitrogen), and antibiotics (Invitrogen). For in vitro experiments, cells were seeded onto the various treatment modalities at a density of 10,000 cells in 24 well culture plates (Corning, New York, NY, USA) for cell proliferation experiments and 50,000 cells per well in 24-well plates for real-time PCR, ALP assay and alizarin red experiments. For experiments lasting longer than five days, medium was replaced twice weekly.

### 2.2. ELISA Protein Release of rhBMP9 from HA

To determine the quantity of rhBMP9 to be released over time from HA, ELISA quantification assay was utilized. Since HA is delivered in a liquid/gel system and rapidly densifies and forms a cross-linking network within a few minutes, HA was delivered simultaneously with rhBMP9, mixed and allowed to densify for 5 min prior to future investigation. Briefly, after the coating the bottom of 24-well cell culture dishes with 100 ng/mL of rhBMP9 and HA, dishes were placed at 37 °C in a shaking incubator (E&K Scientific Products, Santa Clara, CA, USA). Over time, the remaining PBS solution containing unattached rhBMP9 protein was collected, replaced and quantified by a sandwich ELISA (DY3209, range = 15.60–1000 pg/mL, R&D Systems) for the remaining amount of rhBMP9 protein released from HA according to manufacturer’s protocol. Subtraction of total coated protein from the amount of un-adsorbed protein was used to determine the amount of growth factor remaining with HA as previously described [26]. In order to determine the quantity of rhBMP9 protein being released from HA over time, HA was soaked in 1 ml of PBS at all time points and samples were collected at various time points including 15, 60 min, 8 h, one, three and 10 days. All samples were quantified in duplicate and three independent experiments were performed.

### 2.3. Proliferation Assay

ST2 cells were seeded in 24-well plates at a density of 10,000 cells per well either (1) control standard tissue culture plastic (TCP), (2) control HA alone and (3) HA with 100 ng/mL of rhBMP9. Cells were quantified using a MTS assay (Promega, Madison, WI, USA) at one, three and five days for cell proliferation as previously described [27]. At desired time points, cells were washed with PBS and quantified using an ELx808 Absorbance Reader (BIO-TEK, Winooski, VT, USA). Experiments were performed in triplicate with three independent experiments for each condition.

### 2.4. ALP Activity Assay

At seven days, alkaline phosphatase activity was investigated using Leukocyte alkaline phosphatase kit (procedure No. 86, Sigma, St. Louis, MO, USA). ST2 cells were fixed by immersing in a citrate-acetone-formaldehyde fixative solution for 5 min and rinsed in deionized water for 1 min. Alkaline dye mixture are prepared by 1 mL Sodium Nitrite Solution and 1 mL fast red violet alkaline solution dissolved in 45 mL of distilled water and 1 mL of Naphtol AS-Bl alkaline solution. Surfaces were then placed in alkaline dye mixture solution for 15 min protected from light. Following 2 min of rinsing in deionized water. All images were captured on a Wild Heerbrugg M400 ZOOM Makroskop (Wild Heerbrugg, Heerbrugg, Switzerland) at the same magnification at the same light intensity and imported onto Image J software (NIH, Bethesda, MD, USA). Thresholding was used to generate percent stained values for each field of view.

### 2.5. Real-Time PCR for Osteoblast Differentiation Markers

Real-time RT-PCR was used to investigate the expression of genes encoding osteoblast differentiation markers. Total RNA was isolated using High Pure RNA Isolation Kit (Roche, Basel, Switzerland) at three and 14 days. Primer and probe sequences for genes encoding collagen1α2 (COL1a2), alkaline phosphatase (ALP), osteocalcin (OCN), and glyceraldehyde 3-phosphate dehydrogenase (GAPDH) were fabricated with Primer sequences according to Table 1. Reverse transcription was performed with Transcriptor First Strand cDNA Synthesis Kit (Roche). Real-time RT-PCR was performed using Roche FastStart Universal SYBR Green Master and quantified on an Applied Biosystems 7500 Real-Time PCR Machine (Biosystems, Life Technologies Corporation, Carlsbad, CA, USA). A Nanodrop 2000c (Thermo, Wilmington, DE, USA) was used to quantify total RNA levels. All samples were assayed in duplicate with three independent experiments were performed. The ∆∆Ct method was used to calculate gene expression levels normalized to total RNA values and calibrated to control samples.

### 2.6. Alizarin Red Staining

Alizarin red staining was performed to determine the presence of extracellular matrix mineralization. After 14 days, cells were fixed in 96% ethanol for 15 minutes and stained with 0.2% alizarin red (Alizarin red S; Sigma) solution in water (pH 6.4) at room temperature for 1 h as previously described [13]. Alizarin red quantification was performed using images captured on a Nikon D610 camera with a Heerbrugg M400 ZOOM microscope (Wild Heerbrugg). Image J software was used to quantify data with the same threshold values for all analyzed.

### 2.7. Statistical Analysis

All experiments were performed in triplicate with three independent experiments for each condition. Mean and standard error (SE) were analyzed for statistical significance using one-way analysis of variance with Tukey posy hoc test (*, *p*-values < 0.05 was considered significant) for ALP and alizarin red experiments, and two-way analysis of variance with Tukey post hoc test (*, *p*-values < 0.05 was considered significant) for proliferation assay and real-time PCR experiments, by GraphPad Prism 6.0 software (GraphPad Software, Inc., La Jolla, CA, USA).

## 3. Results

### 3.1. Surface Characteristics of HA Scaffolds and Ability to Hold and Release rhBMP9 over Time

**Figure 2:**
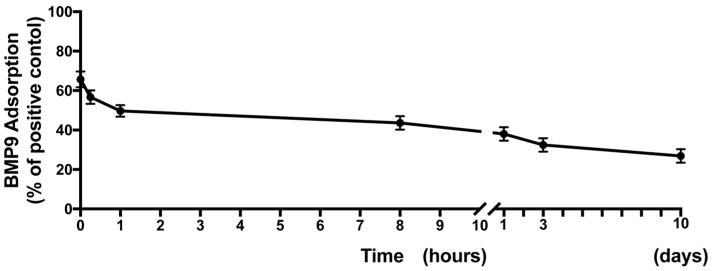
rhBMP9 incorporation into HA scaffolds and its release over time at 0, 15, 60 min; 8, 24 h; 3 and 10 days as quantified by ELISA. An initial burst of released HA was observed between 0 and 1 h. Thereafter, HA was able to efficiently hold rhBMP9 within its scaffold which was thereafter slowly released into the surrounding media over a 10-day period.

In a first set of experiments, the morphological three-dimensional features of HA scaffolds were investigated via SEM (Figure 1). It was first found at low magnification that HA displayed a somewhat wavy surface architecture with many pits and grooves found on its surface (Figure 1A). Higher magnification imaging revealed the cross-linked pattern of the HA scaffolds (Figure 1B,C). Thereafter, rhBMP9 was incorporated into the HA and the quantity of total BMP9 released over a 10-day period was observed (Figure 2). It was first found that at early time points, HA maintained a 70% concentration of BMP9 within its scaffold when compared to original loading (Figure 2). rhBMP9 was then released from 70% to 40% within the first 24 h, and thereafter was slowly released up to a 10-day period (Figure 2). At 10 days, HA contained roughly 35% of the initial content of rhBMP9, demonstrating its ability to hold and slowly release this growth factor over time in a controlled manner (Figure 2).

### 3.2. Effect of HA Alone and in Combination with rhBMP9 on ST2 Cell Proliferation 

**Figure 3:**
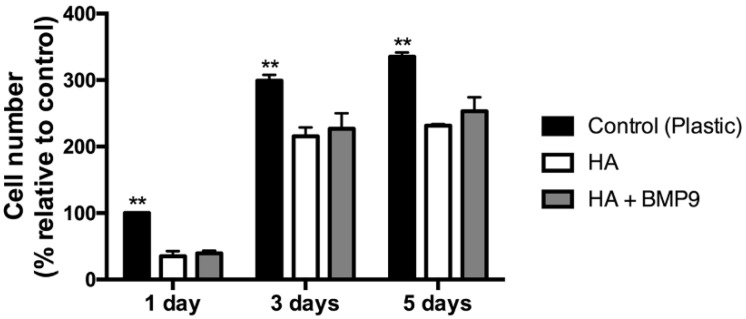
Proliferation assay at one, three and five days post-seeding of ST2 cells seeded on (1) control tissue culture plastic (TCP), (2) control HA and (3) HA in combination with 100 ng/mL of rhBMP9. The highest cell numbers were found on control TCP with no differences observed between HA with/without rhBMP9 (** denotes significantly higher than all other treatment modalities, *p* < 0.05).

Both the regenerative potentials of HA and rhBMP9 were then investigated for the ability for progenitor osteoblasts to proliferate (Figure 3). While it was found that all cells grew on TCP, HA and HA scaffolds with rhBMP9, significantly higher cell numbers were observed on control TCP (Figure 3). Therefore, cells seeded directly to TCP attached and proliferated the fastest when compared to HA or HA + rhBMP9 (Figure 3). The incorporation of rhBMP9 into HA scaffolds did not significantly influence cell proliferation at one, three or five days post-seeding when compared to HA alone (Figure 3).

### 3.3.Effect of HA on ST2 Cell Differentiation When Combined with rhBMP9

**Figure 4:**
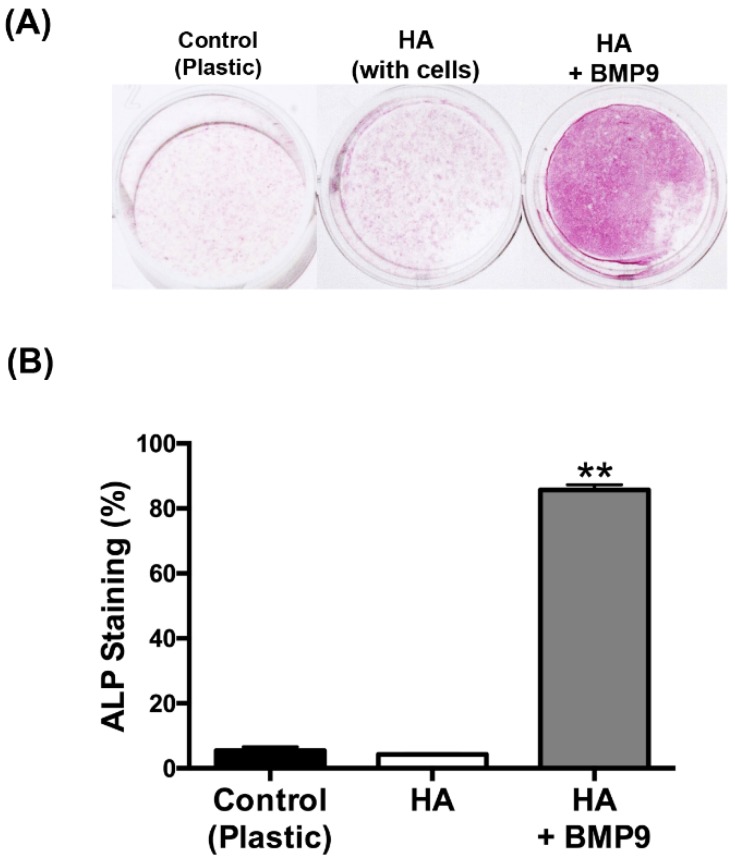
(**A**) Alkaline phosphatase staining at seven days of ST2 cells seeded on (1) control tissue culture plastic (TCP), (2) control HA and (3) HA in combination with 100 ng/mL of rhBMP9. (**B**) rhBMP9 significantly and markedly enhanced ALP staining when compared to both control TCP and control HA groups (** denotes significantly higher than all other treatment modalities, *p* < 0.05).

**Figure 5:**
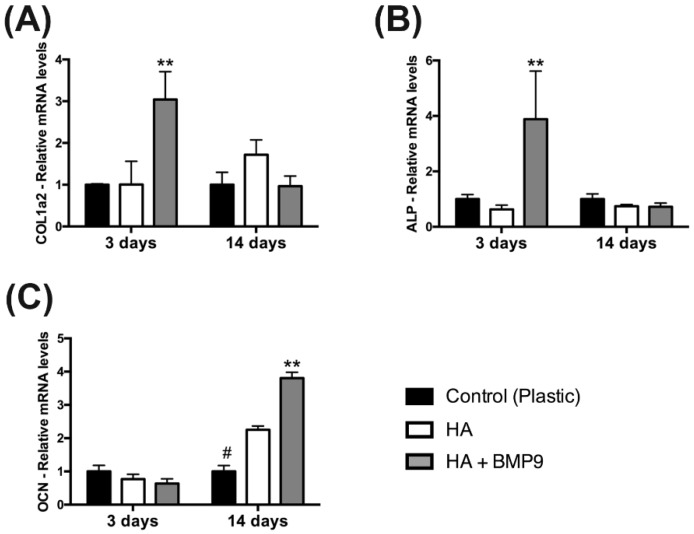
Real-time PCR of ST2 cells seeded on (1) control tissue culture plastic, (2) control HA and (3) HA in combination with 100 ng/mL of rhBMP9 for genes encoding (**A**) collagen 1α2 (COL1a2), (**B**) alkaline phosphatase (ALP) and (**C**) osteocalcin (OCN) at three and 14 days post-seeding (# denotes significantly lower than all other modalities, *p* < 0.05; ** denotes significantly higher than all other treatment modalities, *p* < 0.05).

**Figure 6:**
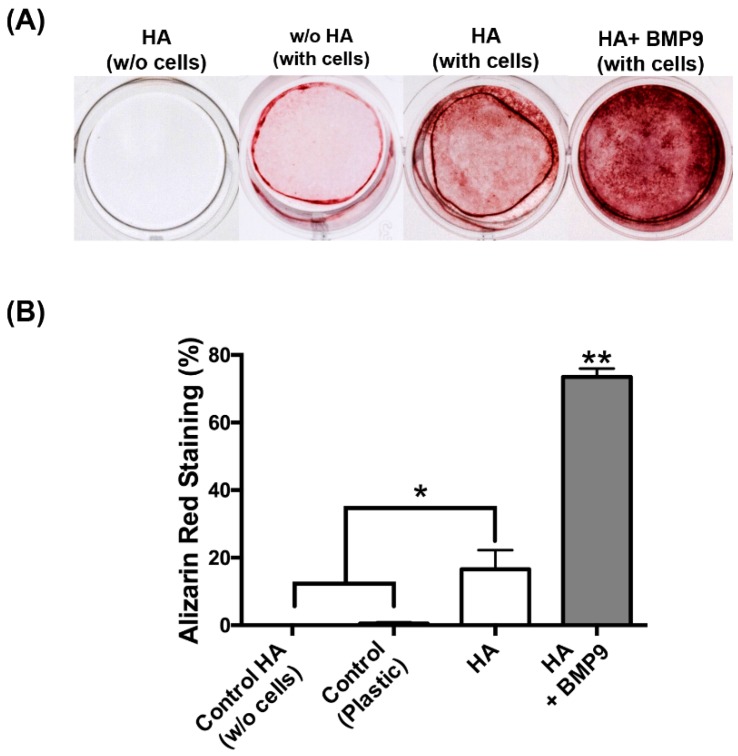
(**A**) Visual representation of alizarin red stained of (1) negative control HA without cells, (2) control tissue culture plastic (TCP), (3) control HA and (4) HA in combination with 100 ng/mL of rhBMP9 at 14 days post-seeding. Note the intensity of red staining on HA scaffolds with BMP9 in comparison to control TCP and HA alone groups. (**B**) Quantified data of alizarin red staining from color thresholding software (* denotes significant difference, *p* < 0.05; ** denotes significantly higher than all other treatment modalities, *p* < 0.05).

Thereafter, the effects of HA with/without rhBMP9 were tested on osteoblast differentiation (Figure 4, Figure 5 and Figure 6). It was first found that while HA had no influence on ALP activity at seven days post-seeding, its combination with rhBMP9 markedly increased ALP activity (Figure 4). Thereafter, real-time PCR was utilized to investigate genes encoding COL1a2, ALP and OCN (Figure 5). It was first found that COL1a2 levels were significantly higher in groups containing both HA and rhBMP9 (three-fold increase) when compared to either control TCP or control HA (Figure 5A). No difference was observed between TCP or HA and no significant differences were observed between all groups at 14 days (Figure 5A). Similarly, ALP was increased approximately four-fold when compared to control TCP and control HA groups at three days, yet no differences between groups were observed at 14 days (Figure 5B). Lastly, OCN expression (a late marker for osteoblast differentiation) demonstrated no changes in gene expression after three days; however, at 14 days HA alone induced a two-fold significant increase and HA + rhBMP9 induced a four-fold increase that was significantly higher than that of all other groups (Figure 5). Lastly, alizarin red staining was utilized to determine the mineralization potential of HA and rhBMP9 (Figure 6). It was found that HA alone had the ability to induce osteoblast differentiation whereas its combination with rhBMP9 further induced over a four-fold increase in alizarin red staining (Figure 6).

## 4. Discussion

During osteogenesis, bone formation occurs in a well-orchestrated process when pluripotent mesenchymal stem cells differentiate into pre-osteoblasts instead of serving as progenitors for myocytes, adipocytes or chondrocytes [28]. These pre-osteoblasts (such as the ST2 cells utilized in this study) are then able to differentiate into osteoblasts based on their extracellular matrix environment should they receive the appropriate cues to fully differentiate into mature bone-forming osteoblasts [28,29,30]. Noteworthy, BMPs in general have played an important role in regulating the tissue pool of pre-osteoblasts and help to significantly induce their subsequent differentiation towards producing mature bone [31,32].

A variety of regenerative agents have been brought to market in recent years to fill the large number of bone defects which are a result of trauma, inflammation, congenital disease or fracture [31,32,33,34]. Among the number of available options, autogenous bone has been considered the gold standard due to its excellent combination of three characteristic properties of bone grafting materials including osteoconductivity, osteoinductivity and osteogenecity [25,35]. Despite this, various reports from the literature have often commented on the shortcomings of autogenous bone grafts which include limited supply, donor-site morbidity, additional surgical time and costs [36]. Thus, the aim of tissue regeneration is therefore to create a biocompatible artificial replacement material, such as metals, xenografts, allografts or synthetic alloplasts, to replace the need to harvest a limited supply of autogenous sources.

More recent research has shown that a variety of hydrogels and nanogels are able to serve as ideal scaffolds for tissue regeneration by either carrying live progenitor cells or growth factors within their matrix [30,37,38,39,40]. Furthermore, the properties of various gel-like biomaterials, such as a the cross-linked gel carrier system utilized in this study, are able to facilitate new bone formation as quantified either in vitro or in vivo [21,22,23,24]. One of the advantages of HA is that it is naturally found in many connective tissues and thus serves as a completely inert biocompatible material [21,22,23,24]. Therefore, in light of these positive previous studies confirming the ability for HA alone to support osteogenesis, the aim of the present study was to utilize cross-linked HA as a possible carrier system for growth factors for bone tissue engineering.

Of the list of possible growth factors, BMPs are the most bone-inducing as at least 15 types of BMPs have been identified in humans [31,32]. Previous analysis of the osteogenic activity of 14 types of human BMPs utilizing transfection experiments (i.e., BMP-2 to BMP-15) found that BMP-2, BMP-6, and BMP-9 were the most potent factors promoting osteogenic differentiation both in vitro and in vivo [5,6,10,11]. Kang et al. first reported that an adenoviral vector that transduced osteoblast progenitor cells will highly and efficiently continue to produce biologically active BMPs inside mammalian cells favoring mature osteoblast differentiation [5]. Under these bone-inducing conditions, cells transfected with adBMP-2 showed a 169-fold increase in the activity of the early osteogenic marker ALP over reference cells transfected with GDP, while cells transfected with adBMP-9 showed a 273-fold increase in the activity of ALP [5]. Thus, it was concluded that adBMP-9 mediated greater osteoblast differentiation when compared to adBMP-2 [5]. Unfortunately, these results have since not been translated to any viable clinical means as the use of adenovirus transfection experimentation (gene therapy) is still not approved for routine generative procedures by the FDA. Alternative strategies therefore remain necessary.

The only FDA-approved strategy utilizing growth factors has been via the use of recombinant sources, where rhBMP2 has been by far the most heavily investigated growth factor for bone regeneration [41,42,43,44]. Despite its widespread use, a number of drawbacks, including a short biological half-life, lack of a matrix which allow its controlled and sustained release, and inability of the recombinant molecule presentation after implantation to mimic normal in vivo protein folding by a BMP-producing cell, have all been reported [45]. Further reported problems include a multitude of secondary side effects including retrograde ejaculation, antibody formation, postoperative radiculitis, postoperative nerve root injury, ectopic bone formation, vertebral osteolysis/edema, dysphagia and neck swelling, hematoma formation, interbody graft lucency, and wound healing complications [46]. Therefore, there remains great interest to find a growth factor that is equally as or more osteopromotive than rhBMP2, yet does not necessitate such high supra-physiological doses causing an abundance of secondary effects.

Recently, our group compared the osteogenic potential of rhBMP9 to rhBMP2 and found that rhBMP9 markedly and significantly induced higher osteoblast differentiation as assessed by ALP staining, alizarin red staining and real-time PCR for osteoblast differentiation markers [12,13]. Furthermore, we observed that rhBMP9 even at low concentrations of 10 ng/mL was able to significantly induce over a two-fold increase in osteoblast differentiation when compared to rhBMP2 at a high concentration of 100 ng/mL, representing higher osteogenic potential even at 10-times-lower doses [12,13]. Therefore, and based on these positive results, the future aim for our group is to find a suitable biomaterial carrier system capable of loading and releasing rhBMP9 over extended periods of time to maximize potential new bone formation in future studies.

HA has been utilized as a biomaterial in many medical applications due to its high biocompatibility with host tissues and its ability to act as a dermal augmentation filler, an adhesion barrier or a drug delivery carrier [16,47,48,49]. Its cross-linked feature has been shown to improve the overall mechanical properties of the scaffold material, further stimulating osteogenesis both in vitro and in vivo [21,22,23,24]. In the present study, we found that HA was able to efficiently load rhBMP9 into its scaffold matrix which was released over a 10-day period (Figure 2). One of the limitations to this experiment was the fact that growth factor release was only characterized up to 10 days. Future research investigating its release prolife over longer periods is necessary. Nevertheless, it was shown that HA alone was able to upregulate the mineralization potential of osteoblasts and certain mRNA levels of osteoblast differentiation markers, confirming its ability to induce osteoblast differentiation when used alone (Figure 5 and Figure 6). More markedly, when HA was combined with rhBMP9, a significant increase in all tested differentiation parameters was observed including ALP activity, real-time PCR for genes encoding COL1a2, ALP and OCN, as well as alizarin red staining. While the results from the present study were not compared directly to rhBMP2 as in our group’s previous publication utilizing rhBMP9, similar trends were observed, again confirming the efficiency for rhBMP9 to act as a strong and potent inducer of osteoblast differentiation. Furthermore, HA seems to act as an ideal biomaterial and carrier system for bone regeneration as it is viscous in nature but allows for three-dimensional growth of incoming cells within its scaffold. Therefore, as a next strategy our group plans to investigate this combination in an animal model, to determine to what extent the regenerative potential of rhBMP9 in combination with cross-linked HA scaffolds serves as an inducer of new bone formation.

## 5. Conclusions

The findings from the present study demonstrate that HA is a purely biocompatible material able to induce the differentiation of osteoblasts. Furthermore, it was shown that the cross-linking HA utilized in this study was able to hold and release rhBMP9 over a 10-day period which significantly further improved osteoblast differentiation by demonstrating higher levels of ALP activity, alizarin red staining and mRNA levels of osteoblast differentiation markers. The results from this study show great promise for cross-linking HA to be potentially utilized not only as a regenerative agent for bone formation, but also as a carrier system capable of holding growth factors such as rhBMP9 or potentially a variety of others for the treatment of other various medical conditions. Future animal studies are necessary to determine the essential role of the carrier system and its importance in tissue regeneration utilizing growth factors in vivo.

## Figures and Tables

**Table 1:** PCR primers for genes encoding Runx2, ALP, COL1a2, BSP, OCN and GAPDH.

Gene	Primer Sequence
mCOL1a2 F	GAGCTGGTGTAATGGGTCCT
mCOL1a2 R	GAGACCCAGGAAGACCTCTG
mALP F	GGACAGGACACACACACACA
mALP R	CAAACAGGAGAGCCACTTCA
mOCN F	CAGACACCATGAGGACCATC
mOCN R	GGACTGAGGCTCTGTGAGGT
mGAPDH F	AGGTCGGTGTGAACGGATTTG
mGAPDH R	TGTAGACCATGTAGTTGAGGTCA
